# Effects of socioeconomic status on risk of ischemic stroke: a case-control study in the Guangzhou population

**DOI:** 10.1186/s12889-019-6998-4

**Published:** 2019-05-28

**Authors:** Siping Wang, Binyan Shen, Meiting Wu, Ciyu Chen, Juan Wang

**Affiliations:** 1 School of Nursing, Guangdong Pharmaceutical University, No. 283 Jianghai Avenue, Haizhu District, Guangzhou, Guangdong China; 2 Department of neurology, the First Affiliated Hospital of Guangdong Pharmaceutical University, Guangzhou, Guangdong China

**Keywords:** Socioeconomic status, Ischemic stroke, Risk factor, A case-control study

## Abstract

**Background:**

The association between socioeconomic status (SES) and stroke risk remains controversial around the world. It is not clear that the effect of SES on stroke in China due to the lack of relevant research. We aimed to assess the association between SES and risks of first-ever ischemic stroke in Guangzhou, China.

**Methods:**

Cases were recruited from neurology department in the First Affiliated Hospital of Guangdong Pharmaceutical University during September 2016–October 2017. Age- and sex-matched controls were derived from surgical departments, over the same period. SES was assessed based on education, occupation, and income. Education was divided into ≤6 years, 6–9 years, 10–12 years, and > 12 years. Family monthly income per capita was categorized into ≤¥1000, ¥1001–3000, ¥3001–5000, and > ¥5001. Occupation was stratified into manual, non-manual, no job, and retired. A multivariate logistic regression model was used to determine the association between SES and risk of ischemic stroke.

**Results:**

In total, 347 ischemic stroke patients and 347 controls were recruited, with mean ages of 60.54 ± 13.13 and 60.56 ± 13.07 years, respectively. After adjusting for confounding factors, odds ratio (OR) for 6–9 years of education was 2.63 (95% confidence interval [CI] 1.45–4.75); > 12 years, 2.18 (1.25–3.82) compared with those for < 6 years of education. ORs for the second lowest, third lowest, and highest incomes were 1.96 (1.21–3.15), 4.16 (2.39–7.22), and 2.83(1.25–6.39), respectively, compared with those for the lowest income. ORs for manual workers and non-manual workers were 1.95 (1.23–3.07) and 1.87 (1.05–3.33), compared with individuals without jobs.

**Conclusions:**

Higher SES is positively related with risks of ischemic stroke, explained by cardiovascular diseases and unhealthy lifestyles in Guangzhou, China. Thus, effective strategies such as extensive health education, promoting a healthy lifestyle, screening for risk factors to prevent stroke should be implemented to reduce ischemic stroke incidences among the high SES group.

**Electronic supplementary material:**

The online version of this article (10.1186/s12889-019-6998-4) contains supplementary material, which is available to authorized users.

## Background

Stroke has become a public health problem worldwide and has caused severe disabilities or deaths, leading to heavy economic, social and emotional burden. The incidence of stroke in low-and-middle income countries (LMICs) is reported to be continuously increasing [1]. In 2016, there were 80.1 million prevalent cases of stroke globally, 84.4% with ischemic stroke, and there were 13.7 million new stroke cases [2]. China is one of the largest developing countries worldwide. Though the incidence of stroke had decreased globally, the figure in China was still increasing, which was far more than that in those developing countries [3]. According to the global burden of disease study 2016, China has the highest age-standardized incidences of stroke among developing countries, with 354 per 100,000 person-years, followed by 335 per 100,000 person-years in Latvia [2]. In addition, the prevalence of stroke had increased slightly in China, and urban prevalence was higher than that in rural area [3]. The overall incidence of first-ever stroke increased by 11.9% annually, and 10.7% of the cases were of ischemic stroke in 1992 to 2015 [4]. Previous studies showed that socioeconomic status (SES) is a predictive factor of stroke [5, 6]. However, the association between SES and the risk of stroke is rather unclear. Most studies reported that low SES can increase the risk of stroke [5–8]. Nevertheless, several studies also showed that high SES had an increased risk of stroke, with inverted U-shaped association or no association between them [9–11].

At present, the rapid economic growth and transformation of the urban-rural dual economic structure makes the gap widely between the rich and the poor, which contributed to inequalities of SES in China. According to the China statistical annual, residents’ income was divided into five group, the lowest and the highest per capita disposable income is ¥5958.4 and ¥64,934.0, respectively. People with below the primary school, junior high school, college or above accounted for 30.5, 38.1, 13.9%, respectively. Manual and non-manual accounted for 55.1 and 44.9%, respectively [12]. A series of inequality of socioeconomic status, can bring to individuals’ health inequalities. People with high SES can obtain optimal health resources, have better health behaviors and higher healthcare utilisation [13, 14].

Guangdong is the first big economy and most populous province in China, there are 113.46 million population in Guangdong in 2018, while 406 thousand population in Guangzhou [12]. The imbalance of regional economic development and distribution of population may result in the inequalities of health. Whether SES was significantly associated with the incidence of stroke remains unclear. Besides, the incidence of ischemic stroke (69.9–76.3%) is higher than intracerebral hemorrhage (23.7–30.1%) in China over the past 20 years, which situation is more serious [4]. Therefore, we examined the potential association between SES and risk of ischemic stroke among the Guangzhou population, and came up with some prevention strategies to reduce risk factors and incidences of ischemic stroke among different SES for future.

## Methods

### Study population

This hospital-based case-control study was performed during September 2016–October 2017. Patients with first-ever ischemic stroke were recruited from the neurology department at the First Affiliated Hospital of Guangdong Pharmaceutical University. Controls with non-ischemic stroke were selected from surgical departments, including orthopedics and traumatology departments, over the same period.

The inclusion criteria for cases were as follows: (1) ischemic stroke diagnosed according to the World Health Organization criteria [15], confirmed by brain computed tomography or magnetic resonance imaging; (2) age ≥ 18 years; and (3) presented to the hospital within 7 days after the onset of symptoms. The exclusion criteria for cases were transient ischemic attack; intracerebral, subdural, or subarachnoid hemorrhage; mental illness; cognitive disorder; epilepsy; brain trauma; brain tumor; and spinal cord lesions. The inclusion criteria for controls were (1) patients with non-stroke aged ≥18 years who presented to the hospital within 7 days and (2) sex- and age-matched controls (±3 years) with cases. Exclusion criteria for cases were stroke, mental illness, mental retardation, epilepsy, brain trauma, brain tumor, and spinal cord lesions.

### Questionnaire

A standardized structured questionnaire was designed according to the related international literature, and domestic expert consultations (see Additional file 1). The questionnaire mainly included four dimensions: demographic characteristics, socioeconomic status, lifestyle factors, and cardiovascular disease factors. Information were collected by interviewed face-to-face using a designed structural questionnaire during the inpatient interview in the hospital. The interviewers were the trained graduate students. Before informal interview, we recruited 30 patients in the hospital for pre-investigation according to the inclusion criteria. Then, the reliability and validity of questionnaire were analyzed using SPSS statistic software. The reliability of questionnaire was confirmed, and Cronbach’s α was 0.832, with a good reliability. The Kaiser-Meyer-Olkin (KMO) value was 0.874 and findings of Bartlett’s test of sphericity were significant (*P* < 0.001), with good validity.

### Socioeconomic status

SES was measured with levels of education, income, and occupation, refer to previous studies in China [16]. Because the average level of education of China is 6–12 years in 2017 and the average monthly disposable income of urban residents is about 1143 yuan [12]. To reduce the bias of measure, we used a detailed and multi-level stratified way to measure SES. Therefore, the level of education was classified into four groups: ≤6 years, 6–9 years, 10–12 years, and > 12 years in this study, while the family monthly income per capita was categorized into four groups: ≤¥1000, ¥1001–3000, ¥3001–5000, and > ¥5001. Most previous studies divided occupation into manual and non-manual workers, representing low and high SES, respectively. Nevertheless, occupation was stratified into four groups: manual, non-manual, no job, and retired in this study because of the difficulty in classifying the retirees.

### Vascular risk factors

Previous studies showed that the intermediate variables such as lifestyle factors and cardiovascular disease factors affect association between SES and stroke [1, 6]. Thus, it is necessary to include those variables to investigate the association between SES and stroke. The physical activity, smoking, alcohol consumption, and diet were considered as lifestyle factors. These factors were the important factors in stroke. Physical activity was defined according to the American College of Sports Medicine (ACSM) guidelines [17]. A question was as follows: “Do moderately intense aerobic exercise at least 30 minutes a day/5 times per week?” or “Do vigorous intensity aerobic exercise at least 20 minutes a day/2 times per week?”. If answer “yes”, the individual was considered to have participated in physical activities. Otherwise, it is considered as lack of physical exercise. Smoking was defined as the consumption of > 1 cigarette a day for > 1 year [18]. Previous studies demonstrated that excessive alcohol consumption can increase the stroke risk [19]. According to WHO recommends, excessive alcohol consumption at 40–60 g/d was considered [20]. Thus, alcohol consumption was determined based on the consumption of > 50 g of alcohol per day or 250 g a week for > 1 year in this study. The patient’s diet, including red meat, vegetable, fruit, whole-grain, and tea consumption, was defined as consumption of > 3 days a week for > 1 year. Cardiovascular diseases, such as hypertension, diabetes, hypercholesterolemia, coronary heart disease, and atrial fibrillation, were diagnosed by a clinical physician based on the medical record or medication use before the ischemic stroke.

### Approvals and patient consents

The study was approved by the local ethics committee at the First Affiliated Hospital of Guangdong Pharmaceutical University. The written informed consent in this study was allowed to waiver as follows: the risk to the subject of the study is not greater than the minimum risk, and written informed consent is not required for conduct or procedures in the same circumstances if out of “research” background, such as interview research, email/telephone survey. Therefore, verbal informed consent was provided by all patients or their proxy before being recruited into the study.

### Statistical analysis

In the univariate analysis, one-way analysis of variance for continuous variables and *χ*^2^ test for categorical variables were conducted to determine the difference of demographics, SES, lifestyle and cardiovascular disease factors between the case and control groups. Multivariate unconditional logistic regression model was used to calculate odds ratio (OR) and its 95% confidence intervals (CIs) for the association between SES and ischemic stroke. In this analysis, SES and potential confounders including age and sex, and variables that were significant (*P* value < 0.10) in univariate analysis were included. We adjusted for different sets of covariables to clarify the effects of confounding factors on the association between SES and ischemic stroke. Model 1 was a univariate modeling, Model 2 was adjusted for age and sex, which play an important role in the relationship between SES and risk of ischemic stroke. Model 3 was adjusted for age, sex, smoking, physical activity, red meat consumption, vegetable consumption, fruit consumption, whole-grain consumption, drinking tea, hypertension, diabetes, hypercholesterolemia, coronary heart disease, and atrial fibrillation. A *P* value of < 0.05 was considered statistically significant, and all statistical analyses were performed with SPSS 13.0 software package (SPSS, Inc., Chicago, IL).

## Results

A total of 347 cases with first-ever ischemic stroke and 347 controls were recruited in this study. The mean age of cases was 60.54 ± 13.13 years, men accounted for 59.1%; while the mean age of controls was 60.56 ± 13.07 years, men accounted for 53.3%. Baseline characteristics of stroke patients and controls are shown in Table 1. The difference in age and sex between the case and control groups was not statistically significant. However, the difference with respect to hypertension, diabetes, coronary heart disease, hypercholesterolemia, atrial fibrillation, education, income, occupation, smoking, physical activity, red meat consumption, vegetable consumption, whole-grain consumption, and drinking tea was statistically significant between the case and control groups.

**Table 1:** Baseline characteristics of stroke patients and controls

Variable	Control (*n* = 347)	Case (*n* = 347)	P
	No. (%)	No. (%)	
Mean age (years)	60.56 ± 13.07	60.54 ± 13.13	0.887^*^
Male	185 (53.3)	205 (59.1)	0.126
Smoking	60 (17.3)	128 (36.9)	< 0.001
Alcohol consumption	37 (10.7)	53 (15.3)	0.071
Physical activity	208 (59.9)	70 (20.2)	< 0.001
Red meat consumption	295 (85.0)	320 (92.2)	0.003
Vegetable consumption	331 (95.4)	317 (91.4)	0.033
Fruit consumption	181 (52.2)	158 (45.5)	0.081
Whole-grain consumption	195 (56.2)	122 (35.2)	< 0.001
Drinking tea	175 (50.4)	135 (38.9)	0.002
Hypertension	113 (0.3)	239 (68.9)	< 0.001
Diabetes	45 (13.0)	85 (24.5)	< 0.001
Coronary heart disease	18 (5.2)	35 (10.1)	0.015
Hypercholesterolemia	47 (13.5)	110 (31.7)	< 0.001
Atrial fibrillation	4 (1.2)	343 (98.8)	0.008
Education (years)			
≤ 6	184 (53.0)	105 (30.3)	< 0.001
6–9	98 (28.2)	104 (30.0)	
10–12	46 (13.3)	108 (31.1)	
> 12	19 (5.5)	30 (8.6)	
Income (¥ /month)			
≤ 1000	131 (37.8)	67 (19.3)	< 0.001
1001–3000	135 (38.9)	176 (50.7)	
3001–5000	50 (14.4)	82 (23.6)	
> 5001	31 (8.9)	22 (6.3)	
Occupation			
No job	92 (26.5)	55 (15.9)	0.002
Manual	99 (28.5)	94 (27.1)	
Non-manual	64 (18.4)	78 (22.5)	
Retired	92 (26.5)	120 (34.6)	

*P* values are based on χ^2^ test; * *P* values are based on one-way analysis of variance

ORs of first-ever stroke according to SES indicators at different models of correction on baseline factors are presented in Table 2.

**Table 2:** Odds ratio for patients with different levels of socioeconomic status (SES)

Variable	Model 1	P	Model 2	P	Model 3	P
Education (years)						
≤ 6	1.00		1.00		1.00	
6–9	1.86 (1.29–2.68)	0.001	2.11 (1.76–3.69)	< 0.001	2.63 (1.45–4.75)	< 0.001
10–12	4.11 (2.70–6.26)	< 0.001	4.68 (2.04–5.12)	< 0.001	1.56 (0.82–2.98)	0.174
> 12	2.77 (1.49–5.16)	0.001	3.27 (0.71–2.48)	0.370	2.18 (1.25–3.82)	0.006
Income (¥ /month)						
≤ 1000	1.00		1.00		1.00	
1001–3000	2.55 (1.76–3.69)	< 0.001	2.54 (1.43–3.11)	< 0.001	1.96 (1.21–3.15)	0.006
3001–5000	3.21 (2.03–5.07)	< 0.001	3.23 (2.98–7.35)	< 0.001	4.16 (2.39–7.22)	< 0.001
> 5001	1.39 (0.75–2.58)	0.301	1.33 (1.71–6.28)	< 0.001	2.83 (1.25–6.39)	0.012
Occupation						
No job	1.00		1.00		1.00	
Manual workers	1.59 (1.03–2.46)	0.038	1.50 (0.94–2.40)	0.091	1.95 (1.23–3.07)	< 0.001
Non-manual workers	2.04 (1.27–3.26)	0.003	1.91 (1.13–3.21)	0.016	1.87 (1.05–3.33)	0.033
Retired	2.18 (1.42–3.36)	< 0.001	2.15 (1.37–3.35)	< 0.001	1.05 (0.47–2.37)	0.908

Model 1: a univariate modeling. Model 2: multilevel modeling, adjusted for age and sex. Model 3: multilevel modeling, adjusted for sex and age, smoking, alcohol consumption, physical activity, red meat consumption, vegetable consumption, fruit consumption, whole-grain consumption, hypertension, diabetes, coronary heart disease, hypercholesterolemia, atrial fibrillation

In the univariate model, the ORs were 1.86 (95% CI 1.29–2.68) for those with 6–9 years, 4.11 (95% CI 2.70–6.26) for those with 10–12 years, and 2.77 (95% CI 1.49–5.16) for those with > 12 years of education, which were significant compared with those with education of < 6 years. In model 2, the OR for education of 6–9 years and 10–12 years increased to 2.11 (95% CI 1.76–3.69) and 4.68 (95% CI 2.04–5.12), respectively. In model 3, the ORs of education of 6–9 years and > 12 years of education were 2.63 (95% CI 1.45–4.75) and 2.18 (95% CI 1.25–3.82), respectively.

The ORs of 2.55 (95% CI 1.76–3.69) for those with income of ¥1001–3000 per month, 3.21 (95% CI 2.03–5.07) for those with income of ¥3001–5000 per month, and 1.39 (95% CI 0.75–2.58) for those with income of > ¥5001 per month were significant compared with those with ≤ ¥1000 per month. The ratios of ischemic stroke were changed and significant in the adjusted age- and sex-adjusted model and in the adjusted for all factors model.

When compared with no job, an OR of 1.59 (95% CI 1.03–2.46) for manual workers, 2.04 (95% CI 1.27–3.26) for non-manual workers, 2.18 (95% CI 1.42–3.36) for those who were retired increased the risk of ischemic stroke. After further adjustment for age and sex, the ratios of non-manual workers and retired reduced to 1.91 (95% CI 1.13–3.21) and 2.15 (95% CI 1.37–3.35), respectively. The ratios of 1.95 (95% CI 1.23–3.07) for manual workers and 1.87 (95% CI 1.05–3.33) for non-manual workers were observed in the last multivariate model.

## Discussion

The effect of SES on the risk of stroke is becoming an important public health problem in LMICs, especially in China. The inequality in China’s social and economic development is reflected in the inequality in education, income, occupation and power. Therefore, this study evaluated the impact of SES on ischemic stroke by using education, occupation and income of three classical indicators to measure SES. Our findings showed that high SES increased the risk of ischemic stroke. We also found that there was a greater prevalence of vascular risk factors of patients with ischemic stroke.

Previous studies have shown that low SES increased the risk of stroke, which is inconsistent with our findings. Avendano [9] et al. found that lower socioeconomic status was associated with higher stroke incidence for both education and income at ages 65 to 74 in the USA. Seo et al. [11] reported that the incidences of stroke increased as the income level decreased in those 74 years old and under in Korea nationwide study, whereas there was no difference by income levels in those 75 and over. Avendano and Glymour [21] described that wealth and income were independent predictors of stroke at ages 50 to 64, low wealth and income was associated with increased risk of stroke. Jackson et al. [22] reported that lower education level and non-homeownership is associated with increased stroke risk in mid-aged women from the Australian Longitudinal Study. Honjo et al. [8] found that neighborhood deprivation level can increased the incidence of stroke in Japan. Li et al. [23] revealed that the incidence of ischemic stroke was significantly increased with decreasing income. However, whether potential mechanism also exists in the relationship between SES and stroke is not entirely clear, which may be partially mediated by known risk factors, particularly lifestyle, biological factors, and cardiovascular risk factors [8, 22]. Individuals with low SES were less likely to access healthcare services, more inclined to unhealthy behavior, and more likely to have higher incidence of obesity, diabetes, and hypertension [1, 8, 10].

Avendano et al. [9] found that stroke rates were higher among those with the highest education and income beyond age 75, which was similar to our findings. Xu et al. [24] also revealed that after controlling for important confounding factors, participants in the highest and middle categories of income had elevated prevalence of compared with the lowest category. A significantly elevated prevalence of stroke was found in white collar workers compared to blue collar workers, while no significant relationship was observed with education. There are different potential mechanisms that may explain our findings: high SES was associated with higher risk of ischemic stroke. First, individuals with higher SES were more likely to have lack of physical activity or to smoke. While insufficient physical activity and smoking was the independent risk factors for stroke [25]. Chen et al. [26] found that individuals with high income were linked to longer sedentary time. A study showed that increased physical activity among people with higher SES can lower blood pressure, and improve vascular endothelial function, then lower the incidence of ischemic stroke [27]. Liu et al. [28] reported that the proportion of patients with high education was higher in terms of smoking in males, which was consistent with our findings. Second, those with higher SES showed significantly higher stress level from society or life [29]. No-manual workers had an increasing risk of ischemic stroke, because more stress came from life and work. Stress can increase the risk of stroke by affecting the blood pressure, cerebral endothelium, coagulation, or heart rhythm [30]. The pressures transformed into overeating, and stress-driven eaters tended to more frequently consume high calorie foods such as sausages, hamburgers, pizza, and chocolates than other people [31]. Third, people with high SES had a strong purchasing power, and excessive consumption of meat and high calorie foods were likely to be considered. High calorie food consumption such as those from fast-foods increases as income rises, which could also increase the risk of obesity and cardiovascular diseases [32]. A previous study showed that potential healthy dietary pattern mechanisms to reduce the risk of stroke include aiding in weight and glycemic control and improving vascular function and blood lipid and lipoprotein profiles [33]. It is found that manual workers increased the risk of ischemic stroke in the present study, which is consistent with the results of other studies [34]. Manual workers would relax through smoking and alcohol consumption, replenish energy though high calorie food intake due to high energy consumption. We found that the risk of ischemic stroke increased in the retired people. This may be due to changes from the sleeping schedule, social circle, and psychological status of retirees such as sleep deprivation, insufficient exercise, and loneliness. A study showed that short sleepers or sleep-deprived individuals are the important risk factors for ischemic stroke [35]. Besides, isolated and lonely persons are at increased risk of stroke [36].

China’s social transition make civics adopt more unhealthy lifestyles, leading to an increase in cardiovascular diseases in developed regions [37]. From the individual level, it is not yet realized that the effects of unhealthy lifestyles on stroke. The awareness for healthy check-ups and control of cardiovascular diseases is insufficient. From the national level, stroke prevention and control are insufficient in China. Therefore, we proposed some suggestions on the prevention of stroke for people with high SES in developed regions. On the one hand, citizens need to learn to self-management on health, and improve their knowledge, belief, and behaviors on stroke. In knowledge, the public should realize what is an unhealthy lifestyle, what is a risk factor for stroke, and how to prevent stroke; In belief, the public would believe that a positive preventive measures can prevent or delay the occurrence of stroke; In behavior, the public would take the initiative to change unhealthy lifestyle, regularly monitor your blood pressure, blood sugar and lipids, and have regular physical examination. On the other hand, health education about stroke-related knowledge should be carried out through the hospitals, community, workplace as well as mass media, especially based-community and internet health education.

### Limitations

The main contribution of this study lies in investigate the impact of various indicators of SES on ischemic stroke in the Guangzhou case-control study. The findings could contribute to recognize the effect of high SES on ischemic stroke, propose prevent strategies on stroke for people with high SES. However, our study has several limitations. First, the case-control design of the study could generate a selection bias, but we attempted our best to overcome such potential biases by obtaining information in a strict selection standard, with objective evaluations of risk factors as much as possible. Second, the education, income, and occupation are the main source of bias, but we tried to refer to the standard of the average level of country as the classified standard of them. Each indicator was used a detailed and multi-level stratified way. Above all, an accurate way to measure of socioeconomic status needs to be developed in future. We separately investigated education, income, and occupation, ignored the conjointly relation of them. We controlled these factors through different adjusted model and explained these adjusted factors would affect the association between SES and ischemic stroke, with a reporting bias. So, an accurate way like the structural equation model should be used to better explain the mechanism of SES and ischemic stroke. The measurement of some of lifestyle indicators are somewhat crude, such as diet consumption only considers frequency, and there is no key information about their actual food intake, which may have an impact on the results. Third, the study was performed based on a hospital setting, with a small sample. The stroke patients were from hospital, introducing a selection bias, patients who were not admitted to hospital (very minor or rapidly fatal before seeking medical assistance) might not be included. The controls were derived from hospital, not from the general population without the disease or community in the same areas. Therefore, a larger, multicentric, population-based study is further warranted. Finally, the psychological and healthcare factors as mediating variables that affect the relationship between SES and risk of stroke were not considered before the design.

## Conclusions

This study demonstrated that higher SES can increase the risk of ischemic stroke, there was a greater prevalence of vascular risk factors of patients with ischemic stroke. For the population with high SES, effective health education and self-management on health should be taken to improve the knowledge, belief, and behaviors of stroke prevention, so that people will realize that healthy lifestyle can effectively prevent or control risk factors, as well as reduce the incidence stroke in China.

## Additional file

Additional file 1: Questionnaire (DOC 26 kb)

## Abbreviations

CI: Confidence interval
LMICs: Low-and-middle income countries
OR: Odds ratio
SES: Socioeconomic status

## Authors’ information

SW: Masters’ degree candidate, nurse practitioner, the research direction is chronic disease management. School of Nursing, Guangdong Pharmaceutical University, Guangzhou, Guangdong, China. BS: Masters’ degree candidate, the research direction is chronic disease management. School of Nursing, Guangdong Pharmaceutical University, Guangzhou, Guangdong, China. MW: Masters’ degree candidate, supervisor nurse, the research direction is chronic disease management. School of Nursing, Guangdong Pharmaceutical University, Guangzhou, Guangdong, China. CC: Head nurse, department of neurology, the First Affiliated Hospital of Guangdong Pharmaceutical University, Guangzhou, Guangdong, China. JW: Doctor of Medicine, associate professor, master supervisor, head of the teaching and research section, the research direction is chronic disease management. School of Nursing, Guangdong Pharmaceutical University, Guangzhou, Guangdong, China.
