# Supplementary material for: Effects of socioeconomic status on risk of ischemic stroke: a case-control study in the Guangzhou population
Source: BMC Public Health. 2019 May 28;19:648. doi: 10.1186/s12889-019-6998-4 (PMC6537313; doi:10.1186/s12889-019-6998-4)
Supplement: Supplementary file 1 — Questionnaire (DOC 26 kb) [file 12889_2019_6998_MOESM1_ESM.doc]

**Appendix**

Questionnaire

Dear ladies or gentlemen:

Hello! we are interviewing patients trough questionnaires about their demographic characteristics, socioeconomic status, lifestyle, and vascular risk factors in order to exploring the influence factors of disease and providing corresponding health education. This research has been approved by the local ethics committee at the First Affiliated Hospital of Guangdong Pharmaceutical University. All information only for the use of investigation and research, we ensure the security and privacy of personal information, please feel free to fill in. Please help us to fill out a detailed and beat tick in front of the serial number, be careful not to leak. Thank you for your cooperation and support!

Admission time：__________ Admission number ：__________

**Demographic characteristics**

1. Gender：_____

2. Age：_______

**Socioeconomic status**

1. Average monthly household income

（Average monthly household income= Family monthly income/Number of family members）

①≤¥1000

②¥1001-3000

③¥3001-5000

④≥¥5001

1. Education years

①0-6 years

②6-9 years

③10-12 years

④≥12 years

1. Occupation：

①manual (farmers, workers, soldier, athlete, ect)

②non-manual (manager, officer, professionals, self-employed workers, drivers, ect)

③no job (the unemployed, looking for job, housewife, ect)

④retired

**Lifestyles**

1. Do you smoke?: ( smoke >1 cigarette a day, and last more than one year)
2. yes ②no
3. Do you drink?: (drink >50 g of alcohol per day or 250 g a week, and last more than one year)：
4. yes ②no
5. Do you exercise?: (aerobic exercise at least 30 minutes a day/5 times per week or at least 20 minutes a day/2 times per week)
6. yes ②no
7. Nearly a year of your diet: often, more than three times a week
8. Do you often eat red meat?

①yes ②no

1. Do you often eat vegetables?

①yes ②no

1. Do you often eat fruit?

①yes ②no

1. Do you often eat coarse grain?

①yes ②no

1. Do you often drink tea?

①yes ②no

**Cardiovascular disease**

1. Hypertension：
2. yes ②no
3. Diabetes：
4. yes ②no
5. Coronary heart disease：
6. yes ②no
7. Hyperlipidemia：

① yes ②no

1. Atrial fibrillation：

① yes ②no
